# Roses

**Published:** 1880-12

**Authors:** 


### ROSES.

Ladies would all be benefited by a little exercise in the open air every day. Nothing is more delightful, and conducive to health, than gardening, cultivating flowers, etc.

We have in our garden a bed of "Ever-blooming Roses," obtained of the Innisfallen Green Houses, Springfield, Ohio. For the small expense and easy culture of these roses we can speak with confidence. The gratification one takes in producing anything so beautiful as a bed of these lovely tea-roses is something to be willing to labor for. Send to above firm for catalogue.

Death is not the cruel monster that we deem him. He is one of God's brightest angels sent from Heaven to bring home some loved one of earth. So bright are his robes that their glare would blind us were they not covered with a sable mantle.—*Church Union.*

Politics are so one-sided in Vermont that the people have to fight about church matters to have any fun.

